# Elotuzumab enhances natural killer cell activation and myeloma cell killing through interleukin-2 and TNF-α pathways

**DOI:** 10.1007/s00262-014-1610-3

**Published:** 2014-10-07

**Authors:** Balaji Balasa, Rui Yun, Nicole A. Belmar, Melvin Fox, Debra T. Chao, Michael D. Robbins, Gary C. Starling, Audie G. Rice

**Affiliations:** 1 Abbott Biotherapeutics Corp., Redwood City, CA USA; 2 AbbVie Biotherapeutics Inc., Redwood City, CA USA; 3 Bristol-Myers Squibb, Princeton, NJ USA; 4 Present Address: Dako North America Inc., Carpinteria, CA USA; 5 Present Address: Merck Research Labs, Palo Alto, CA USA; 6 Present Address: Medical Science Liaison, USP Medical – Oncology, Research & Development, Bristol-Myers Squibb Company, 777 Scudders Mill Road, Plainsboro, NJ 08536 USA

**Keywords:** Elotuzumab, Interleukin-2, Lenalidomide, Multiple myeloma, Natural killer cell activation, SLAMF7

## Abstract

Elotuzumab is a humanized monoclonal antibody specific for signaling lymphocytic activation molecule-F7 (SLAMF7, also known as CS1, CD319, or CRACC) that enhances natural killer (NK) cell-mediated antibody-dependent cellular cytotoxicity (ADCC) of SLAMF7-expressing myeloma cells. This study explored the mechanisms underlying enhanced myeloma cell killing with elotuzumab as a single agent and in combination with lenalidomide, to support ongoing phase III trials in patients with relapsed/refractory or newly-diagnosed multiple myeloma (MM). An in vitro peripheral blood lymphocyte (PBL)/myeloma cell co-culture model was developed to evaluate the combination of elotuzumab and lenalidomide. Expression of activation markers and adhesion receptors was evaluated by flow cytometry, cytokine expression by Luminex and ELISPOT assays, and cytotoxicity by myeloma cell counts. Elotuzumab activated NK cells and promoted myeloma cell death in PBL/myeloma cell co-cultures. The combination of elotuzumab plus lenalidomide demonstrated superior anti-myeloma activity on established MM xenografts in vivo and in PBL/myeloma cell co-cultures in vitro than either agent alone. The combination enhanced myeloma cell killing by modulating NK cell function that coincided with the upregulation of adhesion and activation markers, including interleukin (IL)-2Rα expression, IL-2 production by CD3^+^CD56^+^ lymphocytes, and tumor necrosis factor (TNF)-α production. In co-culture assays, TNF-α directly increased NK cell activation and myeloma cell death with elotuzumab or elotuzumab plus lenalidomide, and neutralizing TNF-α decreased NK cell activation and myeloma cell death with elotuzumab. These results demonstrate that elotuzumab activates NK cells and induces myeloma cell death via NK cell-mediated ADCC, which is further enhanced when combined with lenalidomide.

## Introduction

Multiple myeloma (MM) is a disease of malignant plasma cells in the bone marrow characterized by anemia, lytic bone lesions, and elevated M protein in blood or urine and is associated with renal dysfunction [1]. MM represents 1 % of all neoplasms and about 13 % of hematological cancers in the USA. Recent advances in treatment options have improved overall survival in patients with MM [2]. Lenalidomide, approved in combination with dexamethasone for the treatment of MM, exerts anti-myeloma activity by multiple mechanisms, including enhancing immune function [3–12]. Although progress has been made in treating MM, most of these patients will eventually become refractory to current treatment and subsequently relapse. Thus, the development of novel targeted therapies and optimal combination strategies is needed to fill a significant unmet medical need in the treatment of patients with refractory/relapsed MM.

Monoclonal antibodies (mAbs) targeting a variety of myeloma cell surface antigens are currently under clinical investigation. The targets of these mAbs include CD38 [13], CD56 [14], CD138 [15], CD200 [16], HM1.24 [17], and RankL [18]. Other targets of mAbs include antagonists of soluble molecules that support myeloma growth such as interleukin (IL)-6 [19] and vascular endothelial growth factor (VEGF) [20]. These mAbs may exert anti-myeloma activity through a variety of mechanisms, including direct induction of cytotoxicity via apoptosis, complement dependent cytotoxicity, antibody-dependent cellular cytotoxicity (ADCC), antibody-dependent cellular phagocytosis, and as antibody drug conjugates to deliver cytotoxic payloads.

SLAMF7, a member of the immunoglobulin (Ig) gene superfamily, is highly expressed on the surface of MM cells with restricted expression on specific lymphocytes including resting natural killer (NK) cells, NKT cells, CD8^+^T cells, activated monocytes, and activated B cells [21]. Given its highly and almost universal expression on MM cells [22], SLAMF7 was identified as a target of MM therapy. Elotuzumab is a humanized IgG_1_ anti-SLAMF7 mAb under clinical investigation for the treatment of relapsed/refractory newly-diagnosed MM, and high-risk smoldering myeloma [2, 23]. In preclinical models, elotuzumab exerts NK cell-mediated target cell killing via ADCC in vitro and has demonstrated antitumor activity in vivo in established myeloma xenograft models [21, 22, 24, 25]. Elotuzumab also promotes cytotoxicity against SLAMF7-expressing myeloma cells by mechanisms other than ADCC, including direct activation of NK cells [26].

A clinical phase I dose-escalation study in patients with relapsed/refractory MM demonstrated that elotuzumab had acceptable tolerability at doses sufficient to achieve biologically relevant serum concentrations and saturate SLAMF7 on myeloma cells in bone marrow [27]. Recent phase II results in the same patient population reported encouraging tolerability and efficacy of elotuzumab in combination with lenalidomide plus low-dose dexamethasone [28]. Based on these results, phase III trials of elotuzumab with lenalidomide plus low-dose dexamethasone were initiated in patients with both newly-diagnosed (ELOQUENT-1; NCT01335399) and relapsed/refractory MM (ELOQUENT-2; NCT01239797).

The objective of the preclinical studies reported here was to provide a mechanistic understanding and strengthen the rationale of combining elotuzumab with lenalidomide for treating patients with MM. Preliminary evidence suggested that pretreatment of either effector NK cells or target myeloma cells with lenalidomide enhanced elotuzumab-mediated ADCC against myeloma cells [24]. Our studies expanded on these findings in a cell co-culture model to further evaluate the anti-myeloma activity of the combination of elotuzumab with lenalidomide.

## Materials and methods

### Cells and cell lines

An in vitro co-culture model of human peripheral blood lymphocytes (PBLs) and myeloma cells was developed to evaluate the mechanism(s) underlying the activity of the elotuzumab and lenalidomide combination. Blood was collected from healthy adult donors, and peripheral blood mononuclear cells (PBMCs) were isolated by Ficoll-Paque™ PLUS (GE Healthcare) according to the manufacturer’s instructions. Monocytes were depleted from the PBMCs using CD14 microbeads (Miltenyi Biotec, Inc., Auburn, CA) or CD14 positive selection kit (StemCell Technologies), and the preparation was confirmed to be >90 % depleted of monocytes. PBMCs depleted of monocytes (PBL-enriched) were used in the assays.

NK cells were enriched from PBMCs using EasySep^®^ NK cell negative selection kit (StemCell Technologies) and confirmed to be >90 % pure by flow cytometry for CD56. Co-cultures enriched or depleted in CD56^+^ lymphocytes were used in experiments to determine IL-2 production and the frequency of IL-2-producing cells in treated cultures (see below). The SLAMF7/CS1+ myeloma cell line OPM2 was obtained from the DSMZ (German Collection of Microorganisms and Cell Culture). IM-9, LP-1, and L363 cell lines were obtained from ATCC. Lenti-green fluorescent protein (GFP) transfection vector (Invitrogen) was used to generate OPM2-, L363-, IM-9-, and LP-1-GFP cells following the manufacturer’s instructions.

### Reagents

The generation of elotuzumab [humanized antihuman SLAMF7 (CS1) IgG1 mAb] and isotype control human IgG1 mAb MSL109 (cIgG1) was described previously [21]. Lenalidomide was custom synthesized; its structure was confirmed by nuclear magnetic resonance spectrometry and the purity was determined to be 99.8 %. A variant of elotuzumab was generated with an IgG2 backbone and mutations in the Fc region (Elo IgG2M3) as previously described [29]. F(ab′)2 fragments of elotuzumab and MSL109 (cIgG1) were generated through pepsin digestion from intact parental antibodies and were found to be completely devoid of intact IgG and free of endotoxin. Recombinant human IL-2 was purchased from Roche, and tumor necrosis factor (TNF)-α and interferon (IFN)-γ were purchased from R&D Systems. Elotuzumab F(ab′)2 binding to SLAMF7 was functionally confirmed by its ability to inhibit the binding of elotuzumab-FITC to SLAMF7 on myeloma cell lines by flow cytometry.

### In vivo myeloma mouse xenograft model

Female ICRTac:ICR Prkdc^scid^ mice (6–8 weeks of age) were obtained from Taconic Farms. After resting for 3–4 days, mice were inoculated in the lower right flank with 1 × 10^7^ OPM2 cells in RPMI-1640 (HyClone). Caliper measurements were done three times weekly for the calculation of tumor volume as described previously [24], and tumor growth was monitored for a period of 6 weeks. Tumor volume was calculated from length × width × height/2, where length is the longest side of the tumor in the plane of the animal’s back, width is the longest measurement perpendicular to the length and in the same plane, and height is the highest point perpendicular to the back of the animal. Mice with an average tumor size of ~100 mm^3^ were randomized into treatment groups of 8 mice each. Lenalidomide in dimethylsulfoxide (DMSO) at 50 mg/kg was administered intraperitoneally in phosphate-buffered saline (PBS) daily for 5 days/week.

Suboptimal dosing of elotuzumab (1 mg/kg) or MSL109 (cIgG1) (1 mg/kg) in PBS with equivalent concentration of DMSO to that of lenalidomide (50 mg/kg) was administered intraperitoneally twice weekly. Experiments were terminated once tumors reached a size of >2,000 mm^3^. Statistical differences between the treatment groups were determined by *t* test using SAS statistical software. Mean tumor volumes between groups were considered significantly different if *P* ≤ 0.05. All studies were approved by the Institutional Animal Care and Use Committee in accordance with the “Guide for the Care and Use of Laboratory Animals” (National Research Council).

### Immunohistochemistry of xenograft tissues

Xenograft tumors were harvested 24 h posttreatment. Goat anti-NKp46 (R&D Systems) goat antibody and Alexa Fluor^®^ 594 donkey anti-goat IgG (Invitrogen) secondary antibodies were used to detect mouse NK cells in OCT-embedded frozen xenograft sections. Slides were mounted in DAPI mounting medium (Vector Labs), and images taken on a fluorescent microscope (Zeiss Axioskop-2). Three fields per tumor at 400× magnification were used for image analysis by Image-Pro Plus software.

### PBL/myeloma co-culture assays

PBLs (2 × 10^6^/mL) from healthy adult donors were co-cultured with lenti-GFP OPM2 target cells (0.2 × 10^6^/mL) at a 10:1 ratio (1 mL/well) in 24-well, flat bottom tissue culture plates. Antibodies (elotuzumab or cIgG1 MSL109) were used at 20 μg/mL. Lenalidomide was dissolved in DMSO and added to wells at 1 μM. Lenalidomide 10 μM was added to co-cultures used for enzyme-linked immunosorbent spot (ELISPOT) assays. Equimolar concentrations of DMSO were used as a control. Upon addition of all the reagents and cells, the tissue culture plates were incubated for 24–72 h at 37 °C/5 % CO_2_. For blocking studies, neutralizing mouse mAb to human IL-2 (clone 5334; R&D Systems), blocking humanized mAb to CD25 (daclizumab), neutralizing humanized mAb to IFN-γ (HuZAF), neutralizing fully human mAb to TNF-α (D2E7), and blocking mouse mAbs to lymphocyte function-associated antigen (LFA)-1 (clone H155-78; BioLegend) were added at 20 μg/mL. Harvested cells were treated with 2 μM EDTA for 30 min at 37 °C, then pipetted thoroughly, collected into 1.5-mL centrifuge tubes, and spun at 2,000 rpm for 10 min. The supernatants were collected and stored at −80 °C until use for cytokine determination in Luminex assays, which were performed for measuring cytokines and growth factors (IL-2, IFN-γ and TNF-α, IL-6, IL-8, IL-15, IL-10, VEGF, and epidermal growth factor) using Millipore MAP human cytokine kits (Millipore). The cell pellets were suspended in 200 μL FACS buffer, and a 50 μL sample was dispensed for immunostaining.

### Flow cytometry

To assess the activation of CD3^−^CD16^+^CD56^+^ NK cells, cells were stained with CD16 PE (clone 3G8 or B73.1), CD56 PE (clone MY31), and CD3 APC H7 (clone SK7) to identify NK cells, and CD54 APC (clone HA58) and CD25 PEcy7 (clone MA-251) (all from BD Biosciences, San Diego, CA) to assess activation status. Dead cells were gated out using propidium iodide. Lenti-GFP-OPM2 cells were used in the assay to facilitate the gating of myeloma cells from the PBL. To quantify the number of myeloma cells, 30 μL of FITC-QuantiBRITE^®^ beads (Polysciences, Inc.) containing approximately 30,000 beads was added to each tube at the time of staining. Data were acquired on FACSCanto™ (Becton–Dickinson), and acquisition was stopped when 5,000 bead events were acquired. Data were analyzed using FACS DIVA software.

### Cell growth assays

OPM2 cells at 500 cells/well were seeded in 96-well flat bottom tissue culture plates. Titrated doses of soluble or immobilized control IgG_1_ or elotuzumab mAb were added to the wells. Cell viability was measured by exposing cells to Alamar Blue for 2 h at 37 °C. Fluorescence was emitted for excitation at 544 nm, and emission was measured at 590 nm. Relative viability was calculated by dividing the fluorescence of elotuzumab-treated or control IgG-treated samples with that of untreated cells. Assay results represent the reading from triplicate wells. Each assay was done twice, with one representative assay shown.

### IL-2 specific ELISPOT assay

To determine the frequency of IL-2-secreting cells, co-cultures were set up for 48 h. Cells were collected, and residual target cells were removed by positive CD138 microbead selection (Miltenyi Biotec, Inc.). PBMC subtraction assays of T cells or NK cells were performed using positive magnetic bead selection of CD3 or CD56 (StemCell Technologies, Vancouver, Canada). Depletion of cell populations was confirmed by flow cytometry. FACS sorting of CD56^+^CD3^+^ T cells was achieved by labeling the treated PBMC cultures with CD56 PE and CD3 APC antibodies. Ninety-six well Immobilon-P high protein binding plates (Millipore, Hayward, CA) were coated with 5 μg/mL capture antihuman IL-2 Ab (BD Biosciences, San Diego, CA) in PBS, incubated overnight at 4 °C, and blocked with medium containing 10 % FBS for 2 h at room temperature. Total PBMCs were re-suspended in complete medium and plated at 5 × 10^5^ cells per well in triplicate wells and incubated for 24 h at 37 °C. Following incubation, cells were removed by two distilled H_2_0 washes followed by three washes with 0.05 % Tween-PBS. Biotinylated antihuman IL-2 detection Ab (BD Biosciences) was added (2 μg/mL) and then incubated for 2 h at room temperature. After three washes with 0.05 % Tween-PBS, plates were incubated with 100 μL of a 1:1,000 dilution of peroxidase-conjugated streptavidin (Jackson ImmunoResearch, West Grove, PA) for 1 h at room temperature. Plates were washed four times with 0.05 % Tween-PBS and developed in 100 μL AEC substrate (BD Biosciences) for 30 min, and then stopped with two distilled H_2_0 washes (200 μL). Plates were dried overnight, and spots were recorded and quantified by an Immunospot^®^ plate reader (CTL, Shaker Heights, OH).

## Results

### Elotuzumab enhanced antitumor activity in combination with lenalidomide and promoted NK cell recruitment to myeloma xenografts

**Fig. 1:**
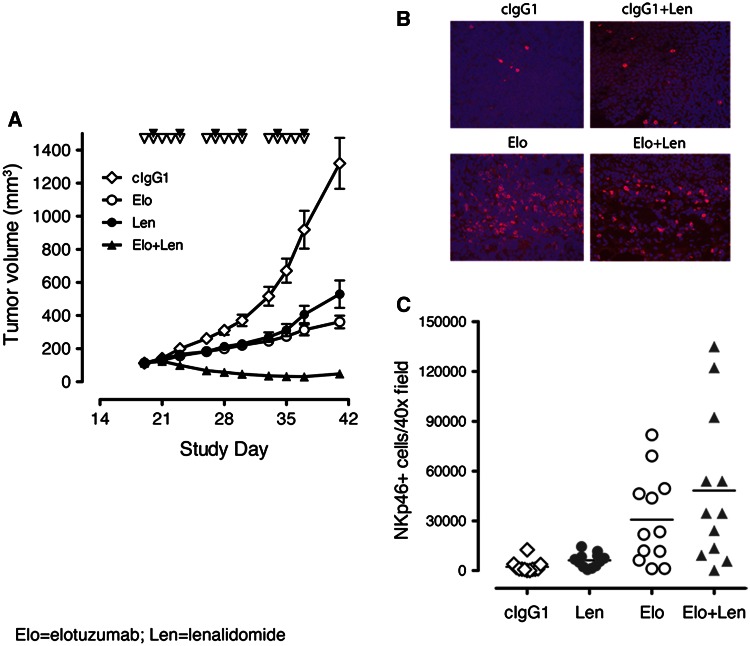
Elotuzumab in combination with lenalidomide enhanced anti-myeloma activity in vivo. **a** Mice with established OPM2 xenograft tumors (average of ~100 mm^2^) were randomized into groups (*n* = 8/group) and treated with control IgG1 (cIgG1), elotuzumab (Elo), cIgG1 plus lenalidomide (cIgG1 + Len), or Elo + Len. *Inverted empty triangles* depict lenalidomide dosing; *inverted filled triangles* depict Elo dosing. Representative data from one of four independent studies are shown. cIgG1 versus Elo, Len, or Elo + Len, *P* < 0.05; Len or Elo versus Elo + Len, *P* < 0.05; Elo versus Len *P* > 0.05. **b** Immunofluorescence staining (magnification 400×) of frozen sections of OPM2 xenograft tumors for NKp46 cell infiltration (*red*) on day 1 post-dosing of cIgG1, Elo, cIgG1 + Len, or Elo + Len. Infiltration of NKp46 cells in OPM2 xenograft tumors was observed in mice treated with Elo or the Elo + Len combination. **c** Image analysis of NKp46 cell infiltration in OPM2 xenografts on day 1 post-dosing. Total amount of NKp46^+^ cell infiltrate (in arbitrary units) of each tumor was measured by image analysis software (Image-Pro Plus). *Each symbol* represents one field of image. Three 400× fields were randomly chosen from each tumor xenograft for image analysis. cIgG1 versus Elo, *P* < 0.01; Elo versus Elo + Len, *P* > 0.05

The combinatorial effects of elotuzumab with lenalidomide in an in vivo setting were investigated in subcutaneous OPM2 xenografts implanted in severe combined immunodeficient (SCID) mice. Inhibition of tumor growth was seen with lenalidomide (50 mg/kg/day at 5 days/week) or a suboptimal dose of elotuzumab (1 mg/kg twice/week for 3 weeks), but established tumors were not fully eradicated. Elotuzumab alone had considerable antitumor activity as compared with cIgG1 control and was similar to lenalidomide alone (Fig. 1a). Elotuzumab plus lenalidomide demonstrated enhanced anti-myeloma activity and suppressed tumor growth to a much greater extent than either single agent.

To test whether the enhanced anti-myeloma activity of the combination of elotuzumab and lenalidomide was the result of increased immune cell infiltration into the xenografts, immunohistochemistry was performed on established OPM2 xenografts to identify the presence of NKp46^+^ NK cells and F4/80^+^ monocyte infiltrates from mice treated with cIgG1, elotuzumab, cIgG1 plus lenalidomide, or elotuzumab plus lenalidomide. Compared with cIgG1, elotuzumab treatment recruited NKp46^+^ NK cells into xenograft tumors, whereas lenalidomide alone did not (Fig. 1b). However, the frequency of NKp46^+^ cell infiltrates was not significantly greater in the OPM2 xenografts of mice treated with elotuzumab plus lenalidomide compared to mice treated with elotuzumab alone when counted per visual field (Fig. 1c). No difference in monocyte infiltrates was observed between any of the treatment groups (data not shown). Furthermore, a variant of elotuzumab with an IgG2 backbone and Fc region mutations (Elo IgG2M3), which abrogated ADCC activity in vitro, did not inhibit tumor xenograft growth and failed to recruit NK cells into the xenografts (data not shown).

### Elotuzumab plus lenalidomide enhanced myeloma cell killing in co-cultures compared to either agent alone

Conventional ADCC assays performed with NK cells or myeloma cells preincubated with lenalidomide were unable to define the combinatorial activity of elotuzumab with lenalidomide in an in vitro setting (data not shown). In order to analyze potential immune mechanism(s) of elotuzumab combined with lenalidomide, a human PBL/myeloma co-culture model was developed (see Materials and methods). Using this model, the effects of elotuzumab and lenalidomide (alone or in combination) could be simultaneously tested on NK cell activation, cytokine production, and myeloma cell killing (determined by myeloma cell counts). Co-cultures were incubated for 48 or 72 h, a time substantially longer than a typical 4-h ADCC assay, which enabled the immunomodulatory effects of lenalidomide to have maximal impact.

**Fig. 2:**
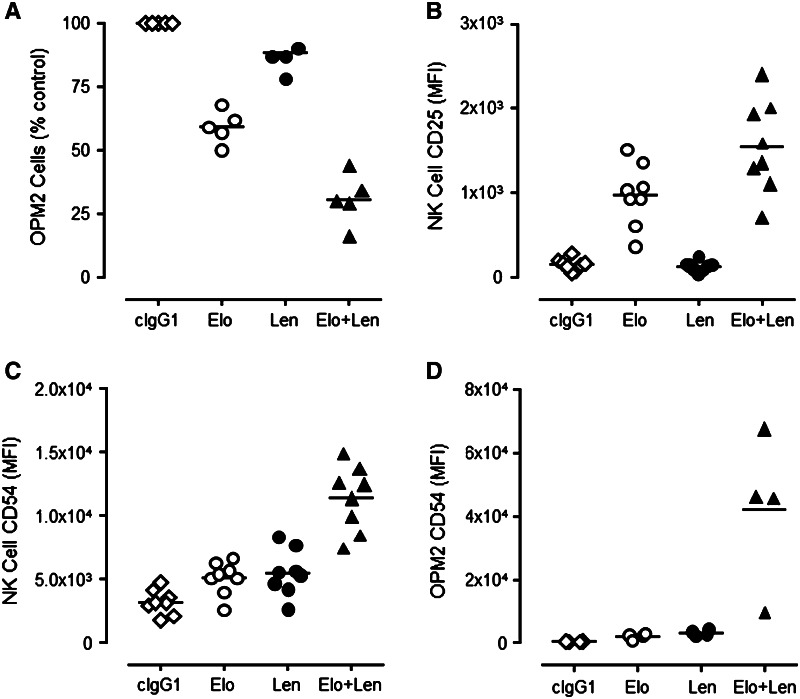
Elotuzumab plus lenalidomide combination enhanced myeloma cell killing and NK cell activation in PBL/myeloma cell co-cultures in vitro. Elotuzumab (Elo) plus lenalidomide (Len) significantly decreased myeloma cell (OPM2) counts compared with Elo (*P* < 0.01) or Len (*P* = 0.01) (*n* = 5) (**a**). Effect of Elo ± Len on CD25 (**b**) and ICAM-1 (**c**, **d**) expression on NK and OPM2 cells (*n* = 4–8). Elo + Len significantly enhanced both IL-2 Rα (*P* < 0.01 vs. Elo; *P* < 0.01 vs. Len) and ICAM-1 (*P* < 0.01 vs. Elo; *P* < 0.01 vs. Len) expression on NK and OPM2 cells more than either agent alone. Elo significantly induced IL-2 Rα (CD25) (*P* < 0.03) and ICAM-1 (CD54) (*P* < 0.01) expression on NK cells more than cIgG1-treated cultures. However, Len had little effect on IL-2 Rα expression (*P* > 0.05), but significantly increased ICAM-1 (*P* < 0.01) expression on NK cells. Neither agent alone had an effect on CD54 expression in OPM2 cells compared with cIgG1

Elotuzumab alone induced significant myeloma cell killing as compared with cIgG1 (Fig. 2a), but the combination of elotuzumab plus lenalidomide significantly decreased the number of OPM2 cells compared with elotuzumab or lenalidomide treatment alone (Fig. 2a). Concomitant with the decrease in OPM2 cells observed in the co-cultures, the combination significantly increased the activation of NK cells as determined by an increase in expression of CD25 (IL-2 receptor α [IL-2 Rα]) (Fig. 2b) and CD54 (ICAM-1, Fig. 2c). Lenalidomide alone had little effect on CD25 expression on NK cells, although it significantly increased CD54 expression. Relative to lenalidomide, elotuzumab alone slightly increased CD25 expression in NK cells, but was comparable in its propensity to increase CD54 expression in NK and OPM2 cells (Fig. 2c, d). Similar results were obtained using other SLAMF7-positive myeloma cells, including IM-9, LP-1, and L363 cells (data not shown).

### Combinatorial activity was dependent on elotuzumab Fc and NK cell LFA-1

**Fig. 3:**
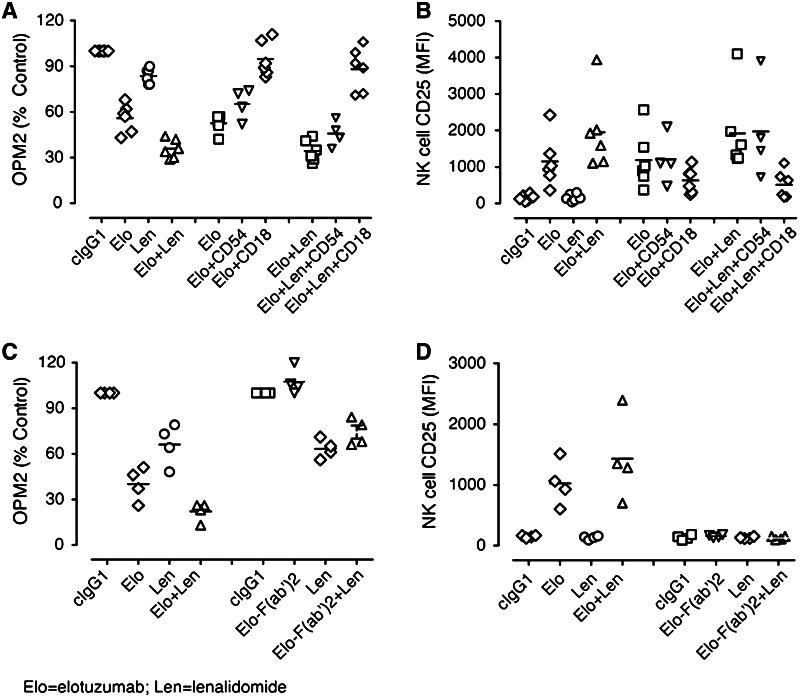
NK cell activation and myeloma killing are LFA-1 and CD16-dependent. **a** Inhibition of myeloma cell killing in PBL/myeloma cell co-cultures in the presence of anti-CD18 mAb (*n* = 6). Addition of anti-CD18 (LFA-1) mAb decreased myeloma cell killing in elotuzumab (Elo) plus lenalidomide (Len) (*P* < 0.01) and Elo (*P* = 0.01)-treated co-cultures more than control IgG1-treated cultures (labeled CD18/54). **b** Inhibition of CD25 upregulation on NK cells in PBL/myeloma cell co-cultures in the presence of anti-CD18 mAb (*n* = 6). Addition of anti-CD18 (LFA-1) mAb decreased CD25 upregulation on NK cells in Elo + Len (*P* < 0.01)- and Elo (*P* < 0.05)-treated cultures more than cIgG1-treated cultures. **c** Requirement of Fc–FcR interaction for Elo-mediated myeloma cell killing (*n* = 4). Elo-F(ab′)2 did not mediate myeloma cell killing as compared to Elo alone (*P* > 0.05), nor did it increase myeloma cell inhibition by Len. **d** Elo-mediated CD25 upregulation on NK cells requires Fc–FcR interaction. Elo-F(ab′)2 did not stimulate the upregulation of CD25 as compared with Elo alone (*P* > 0.05) or in combination with Len

To determine whether NK cell LFA-1 interactions with its ligands are functionally relevant for OPM2 killing, blocking studies with a mAb against CD18 (LFA-1β chain) were performed. Addition of anti-CD18 mAb significantly decreased myeloma cell killing in elotuzumab and elotuzumab plus lenalidomide-treated cultures as compared with control. The addition of an anti-CD54 mAb to elotuzumab and elotuzumab plus lenalidomide-treated cultures also significantly decreased myeloma cell killing, although to a lesser extent than that obtained by blocking with anti-CD18 mAb (Fig. 3a). We also examined whether addition of anti-CD18 mAb was accompanied by inhibition of NK cell activation. The addition of a mAb against CD18 significantly inhibited NK cell activation in elotuzumab and elotuzumab plus lenalidomide-treated cultures (Fig. 3b). The full combinatorial activity of elotuzumab plus lenalidomide in the co-cultures was therefore dependent on LFA-1 binding to one or more of its ligands.

NK cells express two potential binding sites for elotuzumab: SLAMF7 and CD16 (FcγRIIIa). To determine whether elotuzumab-mediated NK cell activation and myeloma cell killing resulted from ligation of SLAMF7 and/or CD16, we tested the effects of Fc deficient elotuzumab F(ab′)2 on NK cell activation and myeloma cell killing in the co-cultures. Soluble elotuzumab F(ab′)2 alone or in combination with lenalidomide failed to mediate myeloma cell killing (Fig. 3c) and induce certain NK cell activation markers (CD25 and CD54 expression) in the co-culture model examined (Fig. 3d). Elotuzumab has been reported to also potentially work through an additional mechanism by bridging SLAMF7 molecules between NK cells and target cells [22, 26]. However, in this specific co-culture system, any bridging of SLAMF7 between effector and target cells was not sufficient to induce NK cell activation and myeloma cell killing. Under the conditions tested, the activity of elotuzumab plus lenalidomide combination was, therefore, dependent on the engagement of elotuzumab Fc to CD16.

### Inflammatory cytokine TNF-α, but not IFN-γ, contributed to combination activity

**Fig. 4:**
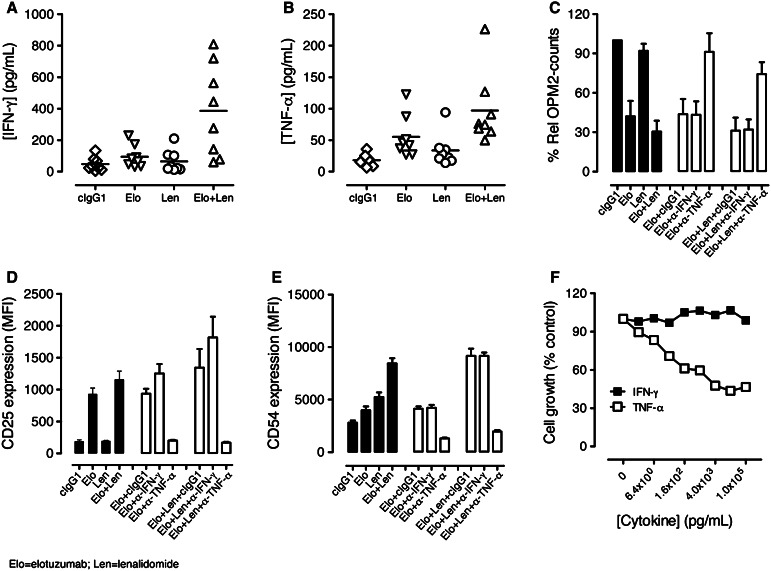
TNF-α and IFN-γ secretion induced by elotuzumab (Elo) in combination with lenalidomide (Len). TNF-α stimulated NK cell activation and myeloma cell killing. (Elo) plus (Len) enhanced **a** soluble IFN-γ (*n* = 8) and **b** TNF-α (*n* = 8) cytokines in the PBL/myeloma cell co-cultures as determined by Luminex assays. Elo + Len significantly enhanced soluble IFN-γ (*P* < 0.05 vs. Elo; *P* < 0.05 vs. Len) and TNF-α (*P* < 0.05 vs. Elo; *P* < 0.01 vs. Len) cytokines as compared with either agent alone. **c** Neutralization of TNF-α (*n* = 4), but not IFN-γ (*n* = 4), significantly inhibited myeloma cell killing (*P* < 0.02 for Elo + anti-TNF-α vs. Elo + cIgG1; *P* < 0.01 for Elo + Len + anti-TNF-α vs. Elo + Len + cIgG1). **d** Neutralization of TNF-α (*n* = 4), but not IFN-γ (*n* = 4), significantly decreased CD25 expression on NK cells (*P* < 0.01 for Elo + anti-TNF-α vs. Elo + cIgG1; *P* < 0.05 for Elo + Len + anti-TNF-α vs. Elo + Len + cIgG1). **e** Neutralization of TNF-α (*n* = 4) but not IFN-γ (*n* = 4) significantly affected CD54 expression on NK cells (*P* < 0.01 for Elo + anti-TNF-α vs. Elo + cIgG1; *P* < 0.01 for Elo + Len + anti-TNF-α vs. Elo + Len + cIgG1). **f** Dose-dependent inhibition of myeloma cell growth in the presence of titrated doses of TNF-α, but not IFN-γ, on myeloma cells as determined in Alamar blue assays on day 4 in vitro. Data are shown from one of two independent experiments

We investigated whether the increased activation of NK cells observed in the PBL/myeloma co-cultures treated with elotuzumab or elotuzumab plus lenalidomide was associated with changes in cytokine production. Elotuzumab alone induced low levels of IFN-γ and TNF-α secretion. Elotuzumab plus lenalidomide significantly enhanced soluble IFN-γ and TNF-α cytokine levels as compared with either agent alone (Fig. 4a, b). To address whether these cytokines contributed to NK cell activation and myeloma cell killing, neutralization experiments were performed with blocking antibodies. Neutralization of TNF-α, but not IFN-γ, significantly inhibited myeloma cell killing (Fig. 4c) and NK cell activation (Fig. 4d, e) in elotuzumab, as well as elotuzumab plus lenalidomide co-cultures.

We also examined whether TNF-α or IFN-γ could exert anti-myeloma activity in the absence of effector cells. Cytotoxicity assays were performed by incubating myeloma cells with TNF-α and IFN-γ. TNF-α, but not IFN-γ, exerted direct dose-dependent anti-myeloma activity (Fig. 4f) at doses that were in the range of soluble TNF-α levels (50–250 pg/mL) produced in the co-cultures. Taken together, these results demonstrate that elotuzumab plus lenalidomide increases soluble TNF-α and IFN-γ levels and that TNF-α contributes to increased NK cell activation and myeloma cell killing. Based on the results of activation assays with NK, PBMC-NK, PBMC-T, and PBMC-monocyte cultures in the presence of elotuzumab or cIgG1, NK cells and monocytes but not T cells contribute to TNF-α production (data not shown).

### Enhanced production and consumption of IL-2 in the presence of elotuzumab

**Fig. 5:**
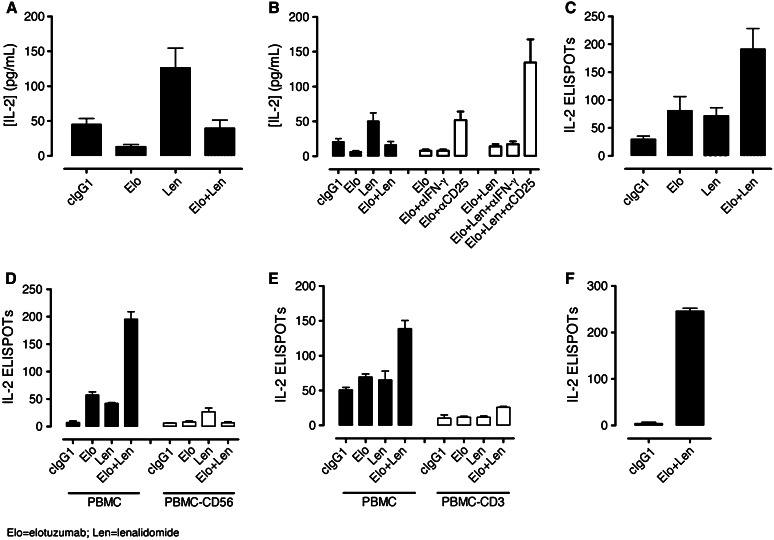
Increased production and consumption of IL-2 stimulated by the combination of elotuzumab (Elo) and lenalidomide (Len). NKT cells are the source of IL-2 in PBL/myeloma co-cultures. **a** Determination of soluble IL-2 levels in co-cultures by Luminex assays (*n* = 17). (Len) plus cIgG1 induced higher levels of IL-2 than cIgG1 cultures (*P* < 0.001). IL-2 levels were decreased in (Elo) (*P* < 0.01 vs. cIgG1) as well as Elo + Len (*P* < 0.01 vs. Len + cIgG1) cultures. **b** Addition of blocking antibodies to IL-2Rα (CD25), but not IFN-γ, increased the accumulation of soluble IL-2 levels in Elo (*P* < 0.01 vs. Elo + cIgG1) or Elo + Len (*P* < 0.01 vs. Elo + Len + cIgG1) cultures (*n* = 6). **c** Determination of IL-2 ELISPOTs in co-cultures (*n* = 9). A higher frequency of IL-2 ELISPOTs was observed in Elo + Len compared with Elo (*P* < 0.01)- or Len (*P* < 0.01)-treated co-cultures. Significant increase in the frequency of IL-2-secreting cells was observed in Elo (*P* < 0.05) and Len (*P* < 0.05) compared with cIgG1 co-cultures. **d** Depletion of CD56^+^ cells significantly decreased IL-2 ELISPOTs in Elo + Len co-cultures (*P* < 0.01 for PBMC vs. PBMC-CD56 lymphocytes) (*n* = 3). **e** Depletion of CD3^+^ cells significantly decreased IL-2 ELISPOTs in Elo + Len co-cultures (*P* < 0.01 for PBL vs. PBL-CD3 lymphocytes) (*n* = 3). **f** Detection of IL-2 ELISPOTs with FACS-sorted CD3^+^CD56^+^ cells from Elo + Len but not from cIgG1-treated co-cultures (*n* = 1)

Using PBMC/myeloma cell co-cultures enriched in CD56^+^ NK cells, we examined whether lenalidomide or the elotuzumab plus lenalidomide combination induced IL-2 production. Consistent with previous reports (4, 30, 31), the levels of IL-2 were higher in lenalidomide plus cIgG1-treated cultures than those treated with cIgG1 (Fig. 5a). In contrast, IL-2 levels were decreased in the cultures treated with elotuzumab, either alone or in combination with lenalidomide. The reduction in IL-2 levels coincided with CD25 expression on NK cells in elotuzumab and elotuzumab plus lenalidomide cultures (Fig. 2b). Based on these observations, we hypothesized that decreased IL-2 levels may be a result of IL-2 consumption by NK cells. To test this hypothesis, we performed IL-2 blocking experiments with anti-IL-2Rα (anti-CD25) mAb. The addition of anti-CD25 mAb to the elotuzumab or elotuzumab plus lenalidomide co-cultures increased soluble IL-2 levels (Fig. 5b). The IL-2 levels were significantly higher in the elotuzumab plus lenalidomide treatment group as compared with either elotuzumab or lenalidomide treatment alone.

To address whether increased soluble IL-2 levels were a result of an increase in the frequency of IL-2—secreting cells in treated co-cultures, IL-2 ELISPOT assays were performed. Both elotuzumab and lenalidomide induced a higher frequency of IL-2—secreting cells compared with cIgG1-treated cultures. Interestingly, the combination of elotuzumab and lenalidomide significantly increased the frequency of IL-2—secreting cells compared to co-cultures treated with either elotuzumab or lenalidomide alone (Fig. 5c). To determine the cellular source of IL-2, PBMC and OPM2 cells were incubated in co-cultures followed by positive depletion of specific lymphocyte subsets and subsequent ELISPOT analysis. Depletion of either CD56^+^ or CD3^+^ cells abolished the IL-2 ELISPOT signal stimulated by elotuzumab and lenalidomide (Fig. 5d, e, respectively).

We hypothesized that a CD3^+^CD56^+^ lymphocyte population was the source of IL-2. To test this hypothesis, T cells (CD3^+^/CD56^−^), NK cells (CD3^−^/CD56^+^), and lymphocytes positive for both CD3 and CD56 from elotuzumab plus lenalidomide or cIgG1-treated co-cultures were sorted by flow cytometry and used in ELISPOT assays. IL-2 ELISPOTs were detected with the CD3^+^/CD56^+^ cells isolated from elotuzumab plus lenalidomide but not from the cIgG1-treated co-cultures (Fig. 5f). Furthermore, IL-2 ELISPOTs were not detected with T or NK cells (data not shown). These findings demonstrate that elotuzumab plus lenalidomide stimulate CD3^+^/CD56^+^ lymphocytes to produce IL-2 and the combination significantly increased both the frequency of IL-2-secreting cells and IL-2 levels.

### IL-2 contributes to the combinatorial activity of elotuzumab and lenalidomide

**Fig. 6:**
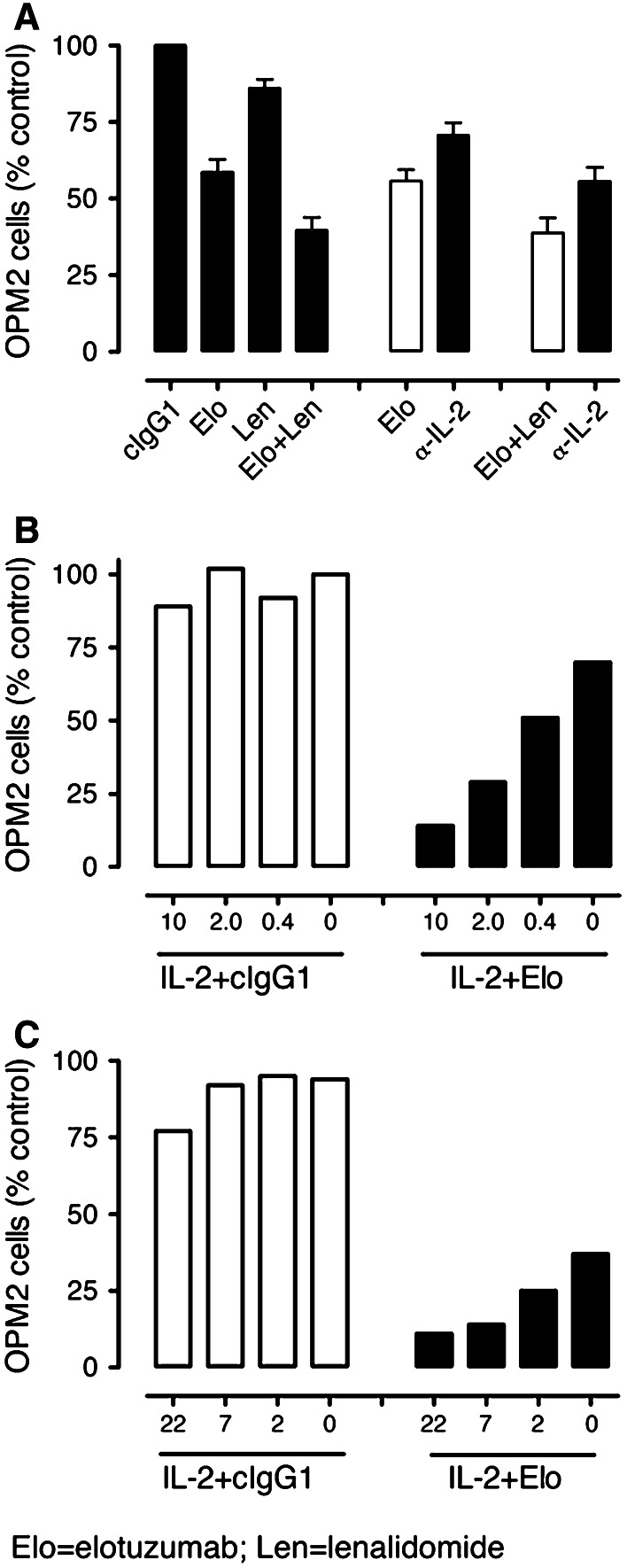
IL-2 contributes to the anti-myeloma activity of elotuzumab (Elo) in combination with lenalidomide (Len). **a** Neutralization of IL-2 with an anti-IL-2 mAb decreases myeloma cell killing in Elo (*P* < 0.05 vs. Elo + cIgG1)- or Elo + Len (*P* < 0.01 vs. Elo + Len + cIgG1)-treated PBL/myeloma cell co-cultures on day 3 (*n* = 7). Enhancement of myeloma cell killing by graded doses of IL-2 (units/mL) in myeloma cell co-cultures with **b** PBL or **c** purified NK cells at 24 h. Representative data from one of the three donors is shown **b, c**

Based on the evidence above that IL-2 production is enhanced but quickly consumed in elotuzumab or elotuzumab plus lenalidomide-treated co-cultures as compared with lenalidomide alone, we hypothesized that IL-2 produced by CD3^+^/CD56^+^ cells contributed to the elotuzumab or elotuzumab plus lenalidomide-mediated NK-dependent myeloma cell killing. To assess the role of IL-2 in myeloma cell killing, a neutralizing mAb to IL-2 was added to co-cultures and compared to isotype control. Addition of the anti-IL-2 mAb to either elotuzumab or elotuzumab plus lenalidomide-treated co-cultures significantly decreased myeloma cell killing compared with control (Fig. 6a).

To provide additional support for the hypothesis that IL-2 contributes to elotuzumab-mediated NK cell-dependent myeloma cell killing, we examined myeloma cell co-cultures either with PBL or with purified NK cells in the presence of elotuzumab and an exogenous source of IL-2. Addition of IL-2 to PBL/OPM2 (Fig. 6b) or NK/OPM2 (Fig. 6c) co-cultures had no effect on NK cell activation or myeloma cell killing. However, addition of IL-2 to elotuzumab in the co-cultures in the presence of either PBL (Fig. 6b) or purified NK cells (Fig. 6c) resulted in dose-dependent myeloma cell killing and NK cell activation as determined by CD25 and CD54 upregulation (data not shown). These findings demonstrated that IL-2 contributed to elotuzumab-mediated NK cell activation and myeloma cell killing in the co-cultures.

## Discussion

In this study, we investigated mechanisms underlying the potential therapeutic benefit of adding elotuzumab to lenalidomide. Evidence suggests that elotuzumab alone has anti-myeloma activity via NK cell-mediated ADCC activity [21, 22, 24, 25], whereas lenalidomide exerts anti-myeloma activity through multiple mechanisms, including direct cytotoxicity and modulation of immune cell activation [4]. Utilizing both in vitro and in vivo models of MM, we found that the combination of elotuzumab with lenalidomide exerted greater anti-proliferative effects than either agent alone. Enhanced inhibition of myeloma cell culture growth with the combination of elotuzumab and lenalidomide was associated with increased NK cell activation, upregulation of adhesion molecules, and stimulation of cytokine production.

We showed that elotuzumab treatment of the xenograft models led to recruitment of NK cells to the tumor and required intact Fc function. NK cell recruitment was not enhanced by treatment with lenalidomide. It is likely that enhanced activity in the model was due to increased NK cell activation through elotuzumab-mediated ADCC, which occurred via recruitment of effector NK cells into SLAMF7/CS1^+^ tumor xenografts and lenalidomide-mediated direct cytotoxic effects against myeloma cells.

The xenograft results were supported by an in vitro co-culture model of PBL and myeloma cells. When combined, elotuzumab and lenalidomide synergized to produce myeloma cell death and NK cell activation across four distinct myeloma cell lines. Moreover, functional activation of the LFA-1–ICAM-1 pathway was observed in our model, with upregulation of ICAM-1 on both NK cells and myeloma cells apparent in treated cultures. The interaction of target cell-expressed ICAM-1 with its co-receptor LFA-1 on NK cells has been shown to play a critical role in adhesion, activating immune synapse formation and target cell killing [30, 31], and antibody blocking of LFA-1/ICAM-1 interactions impairs NK cell cytotoxicity [32]. When an anti-CD18 monoclonal antibody that inhibits the interaction of LFA-1 and ICAM-1 was administered with elotuzumab or elotuzumab in combination with lenalidomide, cell death and NK activity were diminished, suggesting the effect was dependent on the interaction between elotuzumab Fc and NK cell LFA-1.

ADCC mediated by NK cells via Fc–FcR interactions has been demonstrated as a key mechanism of action of elotuzumab activity. However, SLAMF7 is a known mediator of activating signals to NK cells and is also a potentiator of NK target cell interactions via homotypic interactions [33]. Along with the ability of elotuzumab to engage both SLAMF7 and CD16 on NK cells, it has also been shown to potentiate SLAMF7–SLAMF7 homotypic interactions [26], as it binds a membrane proximal epitope of SLAMF7 that is not predicted to be involved in homotypic adhesion. We demonstrated that in the co-culture model evaluated, soluble F(ab′)2 fragments of elotuzumab did not mediate significant activity on NK cell activation alone or in combination with lenalidomide, illustrating the importance of different model systems and assay conditions for investigating the various mechanisms of action of these agents. In the current model system, the CD16-mediated effects of elotuzumab may be dominant over SLAMF7-specific contributions of elotuzumab in mediating NK cell-dependent killing of MM cells.

TNF-α, IFN-γ, and IL-2 are important inflammatory cytokines that influence NK cell activation and may have direct cytotoxic effects on myeloma cells. We showed that TNF-α, but not IFN-γ, exerted direct anti-myeloma cell activity, consistent with previous studies [34, 35]. TNF-α, but not IFN-γ, contributed to NK cell activation mediated by elotuzumab alone or in combination with lenalidomide. The positive effect of lenalidomide on the production of IL-2 by CD4^+^ T cells in the presence or absence of anti-CD3 stimulation has been previously described [4, 36, 37]. IL-2 is also known to play a role in enhancing NK cell cytotoxicity and ADCC against cancer cells [38–40].

Using a sensitive ELISPOT analysis, we determined that elotuzumab plus lenalidomide induced IL-2 production by a CD3^+^CD56^+^ population of lymphocytes that may include NKT cells, but not conventional T cell subsets (CD3^+^/CD56^−^) in our co-culture system. The immune activation phenotype observed in our studies illustrates the complexity of the interaction between elotuzumab and lenalidomide. Aside from the induction of IL-2 in the cultures, the upregulation of CD25, a component of the high affinity IL-2 receptor complex was noted. The functional consequences of blocking the receptor were the accumulation of IL-2 in the co-culture supernatants and reduced killing of myeloma cells in the cultures. Concomitant with the increase in IL-2 pathway components, functional activation of the LFA-1-ICAM-1 pathway was observed, with upregulation of CD54 on both NK cells and myeloma cells.

In conclusion, combining elotuzumab and lenalidomide provides significant enhancement of NK cell function over each agent alone. The enhanced NK cell function is mediated by increased cytokine secretion and upregulation of adhesion and activation molecules on myeloma and NK cells. Enhanced IL-2 secretion by CD3^+^/CD56^+^ cells and production of TNF-α led to enhanced killing of myeloma cells. While these data suggest that the major effect of elotuzumab on NK cell activation is dependent on CD16 engagement via Fc, future studies will focus on mechanisms of NK activation that are independent of Fc:CD16 interactions, i.e., direct SLAMF7 engagement.

Combination therapy is the standard of care for the treatment of MM. A better understanding of the potential mechanisms of action of novel targeted therapies in combination with immunomodulatory and other standard of care agents may lead to an improved rational design of clinical trials, thereby helping to address the unmet medical need in the treatment of MM.

## Abbreviations

ADCC: Antibody-dependent cellular cytotoxicity
DMSO: Dimethylsulfoxide
GFP: Green fluorescent protein
IFN-γ: Interferon-γ
mAb: Monoclonal antibody
MM: Multiple myeloma
NK: Natural killer
IL: Interleukin
PBLs: Peripheral blood lymphocytes
PBMCs: Peripheral blood mononuclear cells
PBS: Phosphate-buffered saline
SLAM: Signaling lymphocytic activation molecule
TNF-α: Tumor necrosis factor-α
VEGF: Vascular endothelial growth factor
